# Photoplethysmographic imaging and analysis of pulsatile pressure wave in palmar artery at 10 wavelengths

**DOI:** 10.1117/1.JBO.27.11.116004

**Published:** 2022-11-10

**Authors:** Jiahong Jin, Jun Q. Lu, Cheng Chen, Ruihai Zhou, Xin-Hua Hu

**Affiliations:** a East Carolina University, Department of Physics, Greenville, North Carolina, United States; b Institute for Advanced Optics, Hunan Institute of Science and Technology, Yueyang, China; c Hunan Institute of Science and Technology, School of Physics and Electronic Science, Yueyang, China; d University of North Carolina, Division of Cardiology, Department of Medicine, Chapel Hill, North Carolina, United States

**Keywords:** *in vivo* imaging, blood vessel, pulsatile pressure wave, light scattering, independent component analysis

## Abstract

**Significance:**

As a noncontact method, imaging photoplethysmography (iPPG) may provide a powerful tool to measure pulsatile pressure wave (PPW) in superficial arteries and extract biomarkers for monitoring of artery wall stiffness.

**Aim:**

We intend to develop a approach for extraction of the very weak cardiac component from iPPG data by identifying locations of strong PPW signals with optimized illumination wavelength and determining pulse wave velocity (PWV).

**Approach:**

Monochromatic *in vivo* iPPG datasets have been acquired from left hands to investigate various algorithms for retrieval of PPW signals, distribution maps and waveforms, and their dependence on arterial location and wavelength.

**Results:**

A robust algorithm of pixelated independent component analysis (pICA) has been developed and combined with spatiotemporal filtering to retrieve PPW signals. Spatial distributions of PPW signals have been mapped in 10 wavelength bands from 445 to 940 nm and waveforms were analyzed at multiple locations near the palmar artery tree. At the wavelength of 850 nm selected for timing analysis, we determined PWV values from 12 healthy volunteers in a range of 0.5 to 5.8 m/s across the hand region from wrist to midpalm and fingertip.

**Conclusions:**

These results demonstrate the potentials of the iPPG method based on pICA algorithm for translation into a monitoring tool to characterize wall stiffness of superficial artery by rapid and noncontact measurement of PWV and other biomarkers within 10 s.

## 1 Introduction

Heartbeat drives pulsatile pressure wave (PPW) through the artery tree to sustain human life.^1^ Imaging of PPW’s spatiotemporal distributions in artery and quantitative analysis allow extraction of vital sign data related to the cardiac activity for monitoring of patients and care of populations at risk.^2^^,^^3^ Most methods for PPW measurement rely on signal detection in the forms of pressure change, acoustic wave echo, Doppler shift, or scattered light.^4^^,^^5^ For example, the method of photoplethysmography (PPG) has long been explored for detection of blood perfusion and oxygenation in peripheral artery from light scattered by tissues.^6^ Pulse oximetry represents the first successful translation of the PPG method into clinic and consumer settings. The PPG method continues to draw active attention for its simplicity and unique capacity to determine heartbeat rate, measure waveforms of PPW, and estimate blood pressures in wearable devices.^7^^,^^8^ Despite these successes, requirement of tissue contact by the PPG method poses considerable risks of pathogen transmission and limits application scenarios like wound monitoring. More importantly, the single- or few-spot nature of PPG method limit its ability to characterize PPW propagation by, e.g., pulse wave velocity (PWV) and spatial distribution for evaluation of arterial stiffness.^2^ It is thus highly desired to leverage PPG’s effectiveness in sensing PPW signals by adding the capacity for spatially resolved measurement.

Imaging photoplethysmography (iPPG) has emerged over the past decade as a noncontact tool for retrieval of heartbeat rate and PPW waveforms.^9^^–^^22^ A video or image stack is acquired to record the spatiotemporal distribution of light backscattered from tissues. A significant challenge is to develop robust algorithms for retrieval of the weak PPW signals with sufficiently high signal-to-noise (SNR) ratios by minimizing strong noises due to slow-moving blood cells, respiration, and unintentional motions. In addition, a useful iPPG method needs to have high spatiotemporal resolutions for accurate extraction of PPW signals and determination of time delays at multiple locations since PPW propagates at a speed on the scales of 1 to 10 m/s.^3^ For a field-of-view (FOV) of 200 mm, spatial resolutions of 0.5 mm or better are needed to carry out meaningful pixel averaging for enhanced SNRs and temporal resolutions of 10 ms or better to resolve time delays within FOV. Furthermore, it is desirable to have an algorithm for retrieval of PPW signals from a monochromatic iPPG stack that permits comparison of different wavelengths to optimize imaging contrast of artery.

In this report, we present a robust algorithm of pixelated independent component analysis (pICA) followed by spatiotemporal filtering for retrieval of waveforms and time delays of PPW from monochromatic iPPG stacks. In a proof-of-principle *in vivo* study with 2 volunteers, the PPW signal distributions in palmar artery were mapped with the new algorithm and compared with analyze the effect of the illumination wavelength band from 445 to 940 nm. We further show that PWV can be derived from the waveform data of 12 volunteers among multiple hand locations at an optimized band of 850 nm with the pICA algorithm.

## 2 Related Work

Various algorithms reported up to date for processing iPPG stack data can be divided into 4 major types by increasing SNRs of the extracted PPW signals. The first was designed to average pixels over selected regions-of-interest (ROIs) within FOV followed by temporal frequency filtering on stacks acquired with rate of 30 frames/s or less.^9^^,^^16^^,^^20^ PPW modulates diffuse reflectance of imaged tissues without measurable time delays in a small ROI and spatial filtering via averaging can effectively suppress pixel intensity variations unrelated to PPW. SNR can be further enhanced by averaging over multiple ROIs of different weighs after frequency filtering.^16^ Pixel averaging used in this type of algorithms, however, comes at the expense of reduced spatial resolution that diminishes the benefit of imaging. To improve, the second type of iPPG algorithms takes the advantage of temporal correlations of single or averaged pixels with a reference signal for isolation of PPW signals. One approach is to use a finger PPG device as the source of reference signal for calculation of its temporal covariance with image pixels that require only monochromatic iPPG stack.^18^ Temporal correlation can also be performed through a homodyne detection scheme for measurement of PPW signals.^11^^,^^17^^,^^19^ The spatially averaged and frequency-filtered pixel intensity was obtained as the reference signal to synthesize maps of PPW signals from the iPPG stack. The SNR improvement at different pixels by homodyne detection relies critically on the SNR of the reference signal which also includes pixels carrying large noise background. Thus, this approach may be of limited SNRs and contrast for waveform analysis at multiple image locations near the artery.

The PPW signals can also be separated in time domain from noise background without utilization of correlation by taking the advantage of statistical independence between them. An independent component analysis (ICA) algorithm has been employed to retrieve the PPW signals from a multispectral iPPG stack acquired by a color camera.^10^^,^^14^^,^^15^^,^^21^ The input signals for the ICA algorithm were formed by the averaged pixel intensities in each of the color channels over a selected ROI and the PPW signal chosen from the ICA output was temporal frequency filtered to obtain waveform data. While the ICA algorithms have been widely accepted for blind source separation, the need for multispectral imaging by a color camera nevertheless limits the ability for optimization of illumination wavelength. A single-channel ICA algorithm has been applied that segments an ROI-averaged monochromatic iPPG stack into multiple blocks in the time domain to form the input signals.^12^ This scheme allows investigations for wavelength optimization but still is of low spatial resolution.

The last type of iPPG algorithm processes a stack acquired with a color camera or multiple near-infrared (NIR) cameras by a skin color model.^13^^,^^22^ The model was derived directly from the image data of a noise-free subject or after processing by a primitive tissue optics model such as the Lambert-Beer law. Then the temporal correlations of pixel intensity among different color channels or wavelength bands were evaluated in a space of dimension given by the color channel number and/or the number of known noise sources, which are assumed to be statistically independent of the PPW signals in the time domain. This approach requires accurate skin color reference data that may not be obtained by the inaccurate model of light scattering, and spatial averaging leads to reduced spatial resolution as discussed above. Finally, it is worth noting that a method of optical metrology by a stereo imaging setup has been applied to determine skin surface vibration near the radial artery in the wrist region.^23^ The waveforms and time delay can be determined from the measured surface vibration in the time domain at three skin locations near the radial artery in the wrist region. This method, however, applies only to locations of palpable skin surface vibration and requires two or more cameras which limit its ability to measure PPW signals in large ROIs.

## 3 Materials and Methods

### 3.1 Acquisition of iPPG Stacks at Different Wavelengths

Monochromatic iPPG stacks have been acquired from healthy volunteers in the Biomedical Laser Laboratory at East Carolina University with no history of cardiovascular diseases and hypertension. This study was performed under a protocol approved by the University and Medical Center IRB of East Carolina University. A volunteer sat upright and was told to keep left-hand stationary on a table for imaging under normal breathing condition. Each iPPG stack was taken over 10 s from the palm side with room light off. Processing of image stacks in time domain required no image alignment. A pulse oximeter (500DL, Vaunn Medical) was worn by every volunteer on a finger of the right hand during imaging for recording of heartbeat rate as the ground truth to validate the rate $f_{h}$ determined from the acquired iPPG stack. Blood oxygenation values were also recorded to monitor health condition and found to be within a normal range of 97% to 99%.

The iPPG system consists of an illumination and an imaging unit with one camera. The illumination unit includes 10 sets of high-power and monochromatic LED arrays with bandwidth of 36 nm on average and peak wavelength $\lambda_{p}$ ranging from 445 to 940 nm. Each set is made of 6 microlensed LEDs arrays on a ring holder to produce a nearly uniform illumination on a target area below the holder with 23 cm in vertical distance and irradiance variation <10% in FOV. Additional information on LEDs is provided in the Supplemental Material. Two monochromatic CMOS cameras were used in this study with the same camera lens of f=8.5  mm in focal length (#58000, Edmund Optics Inc.) and working distance of 635 mm between camera lens and the target area along the vertical direction. One is of a dual-sensor design with 1024×532  pixels per sensor and a maximum frame rate of F=250  Hz (FS-1600D-10GE, JAI Ltd.), which was used to acquire monochromatic iPPG stacks from 2 volunteers in 10 wavelength bands to investigate the effect of wavelength. The other has just one sensor of 640×480  pixels and was employed to take iPPG stacks of 12 volunteers with an LED illumination of $\lambda_{p}=850\;\mathrm{nm}$ with a higher frame rate of F=815  Hz for better temporal resolution. All iPPG images are 8-bit in pixel depth and a cross-polarization configuration was implemented between LED illumination and camera to reduce light backscattered from superficial tissues.

### 3.2 Measurement of Electrocardiogram (ECG) Signals

To validate the extracted PPW signals, we have built an ECG device to measure the rhythm signals of heart from two volunteers during iPPG imaging with $\lambda_{p}=850\;\mathrm{nm}$. The device was developed with a signal processing circuit board (SEN-12650, SparkFun Electronics) based on a dedicated chip (AD8232, Analog Devices). Three electrodes (2560, 3M) are placed on each volunteer’s chest to sample ECG signals at a rate of 8 kHz. The electrode signals are amplified and filtered by the processing circuit and fed to the input of a sound card (SB1570, Creative Technology) in the host computer for digitization. An in-house developed software was used to acquire iPPG data by camera and ECG signals by sound card simultaneously, which allows validation of the cardiac cycles of PPW signals by the ECG signals.

## 4 Algorithms

### 4.1 pICA Algorithm and Two Schemes for Definition of Input Vectors

The proposed pICA algorithm retrieves the PPW signals from an iPPG stack acquired at $\lambda_{p}$. Each image is segmented into two regions of hand and background based on a pixel intensity threshold $I^{\prime }$th determined by histogram.^24^ The intensity of a pixel in the hand region given by $I(x,y;t;\lambda_{p})$ can be expressed as a time vector $I_{n}(x,y;\lambda_{p})$ of following elements after z-score normalization

$$I_{n}(x,y;t;\lambda_{p})=\frac{I(x,y;t;\lambda_{p})-\mu_{I}(x,y;\lambda_{p})}{\sigma_{I}(x,y;\lambda_{p})},$$ (1)

where $\mu_{I}$ and $\sigma_{I}$ are the mean value and variance of $I(x,y;t;\lambda_{p})$ over time, respectively. By averaging $I_{n}(x,y;\lambda_{p})$ over the hand region as $I_{na}(\lambda_{p})$ and Fourier transforming into frequency domain, we determine the heartbeat rate $f_{h}$ as the peak frequency within a band of $\Delta f_{hb}$ between 0.6 and 4.0 Hz for the usual range of human heartbeat rate.

An ICA implementation by joint approximate diagonalization of eigenmatrices (JADE) was chosen for our pICA algorithm to isolate the PPW signal.^25^^,^^26^ ICA assumes that the time dependence of input signals is caused by different sources of statistically independent temporal variations. In our case, the signal sources include PPW driven by heartbeat at a mean rate of $f_{h}$ and, among others, unintentional hand motions and variations of tissues’ optical properties unrelated to PPW. To perform ICA on signals in the form of time vectors, one needs to form a set of $I_{nk}$ representing K-measured input signals with k∈[1,K], and they are assumed to be linearly related to unknown source vectors denoted as $S_{k}$ by an unknown mixing matrix [A] as

$$(\begin{array}{c} I_{n1}\\ \vdots \\ I_{nK} \end{array})=[A](\begin{array}{c} S_{1}\\ \vdots \\ S_{K} \end{array}).$$ (2)

The matrix [A] and source vectors of $S_{k}$ are solved from the underdetermined Eq. (2) iteratively until maximal statistical independence is achieved among the $S_{k}\prime s$, which are named as independent components (ICs) of the input signals. For pICA, a pixel of interest (POI) of vector $I_{n}(x,y;\lambda_{p})$ is designated as the first input vector $I_{n1}$ followed by $I_{n2}$ to $I_{nK}$ of pixels neighboring POI. If a POI is near artery, one IC given by $[A{]}^{-1}(I_{n1},\ldots ,I_{nK}{)}^{T}$ presents the PPW signal characterized by oscillations at $f_{h}$ while other ICs are of variations statistically independent of PPW signals in the time domain. After tests for SNR improvement versus computational cost, we chose K=3 for this study.

Two schemes were used to form the three input vectors for pICA. The first one employs a single-pixel POI at (x,y) in the hand region as $I_{n1}(x,y;\lambda_{p})$. The other two input vectors are obtained as: (1) The pixel intensity averaged over 24 first-neighbor pixels (FNPs) in the two rings surrounding the single-pixel POI for $I_{n2}(x,y;\lambda_{p})$; (2) the pixel intensity averaged over 24 second-neighbor pixels (SNPs) in the third ring surrounding the POI for $I_{n3}(x,y;\lambda_{p})$. Furthermore, we have tested various definitions of $I_{n2}$ and $I_{n3}$ in the first scheme to optimize the image contrasts of synthesized PPW maps related to artery. For example, $I_{n2}$ and $I_{n3}$ can be defined as pixel intensities averaged over two half-hand regions, respectively, that was found to result in significant reduction in PPW map contrasts against the case of FNPs and SNPs. In the following analysis, we term this scheme as PFS for input vectors by POI, FNP, and SNP which was used to determine PPW map of palm at different values of $\lambda_{p}$.

The second scheme is implemented as a zone-averaged one or the PZA scheme for all three input vectors. The first one, $I_{n1}$, is obtained as a time vector of averaged pixel intensity over a square zone of 9×9  pixels centered on a POI selected at (x,y) to increase SNR of the PPW signal. The other two input vectors are given by intensities averaged over 40 pixels in the fifth ring surrounding the POI at (x,y) for $I_{n2}$ and over 48 pixels in the sixth ring for $I_{n3}.$ The PZA scheme was used to determine waveforms of PPW signals and PWV in palmar artery.

### 4.2 Calculation of Pulse Wave Index for Mapping PPW Signals

Among the three pICA output vectors for a POI, the first one denoted as $S_{1}(x,y;\lambda_{p})$ is initially set as the component of PPW signal since it has the largest eigenvalue or strongest temporal correlations with cardiac cycles if the POI is near artery. A test is carried out by Fourier transform of $S_{1}$ into $s_{1}$ in frequency domain to determine a maximum peak frequency $f_{m}$ within the band of $\Delta f_{hb}$. The vector $S_{1}$ is designated as $S_{c}(x,y;\lambda_{p})$ for cardiac component if $|f_{m}-f_{h}|\leq 0.1f_{h}$. Failure of the test leads to reassigning and frequency testing of $S_{2}$ or $S_{3}$. If none of the ICs passes the test, then the vector $S_{k}$ with $f_{m}$ closest to $f_{h}$ is assigned as $S_{c}$. Afterward, $S_{c}(x,y;\lambda_{p})$ is filtered in frequency domain to obtain $S_{\mathrm{cf}}(x,y;\lambda_{p})$ by a finite impulse response (FIR) algorithm to remove frequency components outside the band $\Delta f_{hb}$. The FIR filter is chosen for its ability to produce no phase-shift between $S_{c}$ and $S_{\mathrm{cf}}$,^27^ which enables accurate calculation of time delays in $S_{\mathrm{cf}}$ among multiple POIs.

For POIs close to artery, $S_{\mathrm{cf}}$ and its Fourier transform $s_{\mathrm{cf}}$ yields the PPW signal driven by heartbeat. A pulse wave index $r_{\mathrm{cf}}(x,y;\lambda_{p})$ is defined to quantify the SNR of PPW in the frequency domain and map PPW signal distribution in the hand region. The index is defined as the ratio of total intensities summed in a narrow band as the signal and in a wide band outside the narrow band as noise background or

$$r_{\mathrm{cf}}(x,y;\lambda_{p})=\frac{\overset{N}{\underset{j=-N}{\sum }}s_{\mathrm{cf}}(x,y;f_{m}+j\Delta f;\lambda_{p})}{\overset{W}{\underset{j=-W}{\sum }}s_{\mathrm{cf}}(x,y;f_{m}+j\Delta f;\lambda_{p})-\overset{N}{\underset{j=-N}{\sum }}s_{\mathrm{cf}}(x,y;f_{m}+j\Delta f;\lambda_{p})},$$ (3)

where Δf=F/2d is the frequency stepsize in $s_{\mathrm{cf}}$, d is the minimal integer given by M≤2d≤2M, and M is the total number of images in a stack. For F=250  Hz and M=2500, d=2048 for $s_{\mathrm{cf}}$ with zero padding between M and 2d and Δf=0.0610  Hz. In addition, the total number of frequency components in the narrow and wide bands were set to N=2 and W=12, respectively, after testing different values to optimize contrast of PPW maps. Compared with SNRs defined by the ratio of Fourier components on logarithm scale,^13^^,^^21^ the index defined in Eq. (3) measures PPW signals on a linear scale. It can also be used to determine the gains of SNR by the pICA algorithm as

$$g_{\mathrm{SNR}}=\frac{{\mathrm{SNR}}_{S}}{{\mathrm{SNR}}_{I}}=\frac{r_{\mathrm{cf}}(x,y;\lambda_{p})}{r_{I}(x,y;\lambda_{p})},$$ (4)

where ${\mathrm{SNR}}_{I}=r_{I}(x,y;\lambda_{p})$ is the pulse wave index with $s_{\mathrm{cf}}(x,y;f;\lambda_{p})$ in Eq. (3) replaced by $i_{n1}(x,y;f;\lambda_{p})$ as the Fourier transform of $I_{n1}(x,y;t;\lambda_{p})$ or the first input signal to pICA for either PFS or PZA scheme.

### 4.3 Calculation of Delay Times and PWV

The use of PZA scheme significantly improves the SNR of PPW waveform extracted from iPPG data at a selected POI. We also found that a waveform in terms of inverted $S_{\mathrm{cf}}(x,y;t;\lambda_{p})$ in our study corresponds well to the “conventional” PPW waveform by the PPG method.^28^ One can clearly recognize cardiac cycles and various features in each cycle such as the systolic peak followed by dicrotic notch and diastolic run-off. Consequently, the peak values of an inverted $S_{\mathrm{cf}}(x,y;t;\lambda_{p})$ curve are identified as the systolic blood pressures and the time of “foot” before each peak is used in this study to calculate delay times among different POIs in the same cardiac cycle. To further reduce noise, multiple POIs with negligible time delays in a small region of hand are selected to obtain the mean and standard deviation values of foot-foot time delays between two regions. A region in an iPPG stack can be selected, for example, on wrist or middle of palm, or fingertip near the palmar artery. For a PPW waveform given by inverted $S_{\mathrm{cf}}$ containing C cardiac cycles, we first determine the foot time averaged over the selected POIs in the same region r and cycle c as t(r,c). Then, a delay time between POIs in two hand regions of r and $r^{\prime }$ is obtained as $\tau (r,r^{\prime };c)=t(r^{\prime },c)-t(r,c)$ for PPW propagating from region r to $r^{\prime }$. The delay time is further averaged over c for all cycles as the mean delay time or $\tau_{m}(r,r^{\prime })$ with c∈[1,C]. The PWV values between region r and $r^{\prime }$ is finally obtained as

$$v(r,r^{\prime })=\frac{d(r,r^{\prime })}{\tau_{m}(r,r^{\prime })},$$ (5)

where $d(r,r^{\prime })$ is the averaged straight-line distance between POIs in region r and $r^{\prime }$.

## 5 Results and Discussions

### 5.1 Retrieval of Palmar PPW Maps by the PFS Scheme with Single-Pixel POIs

**Fig. 1:**
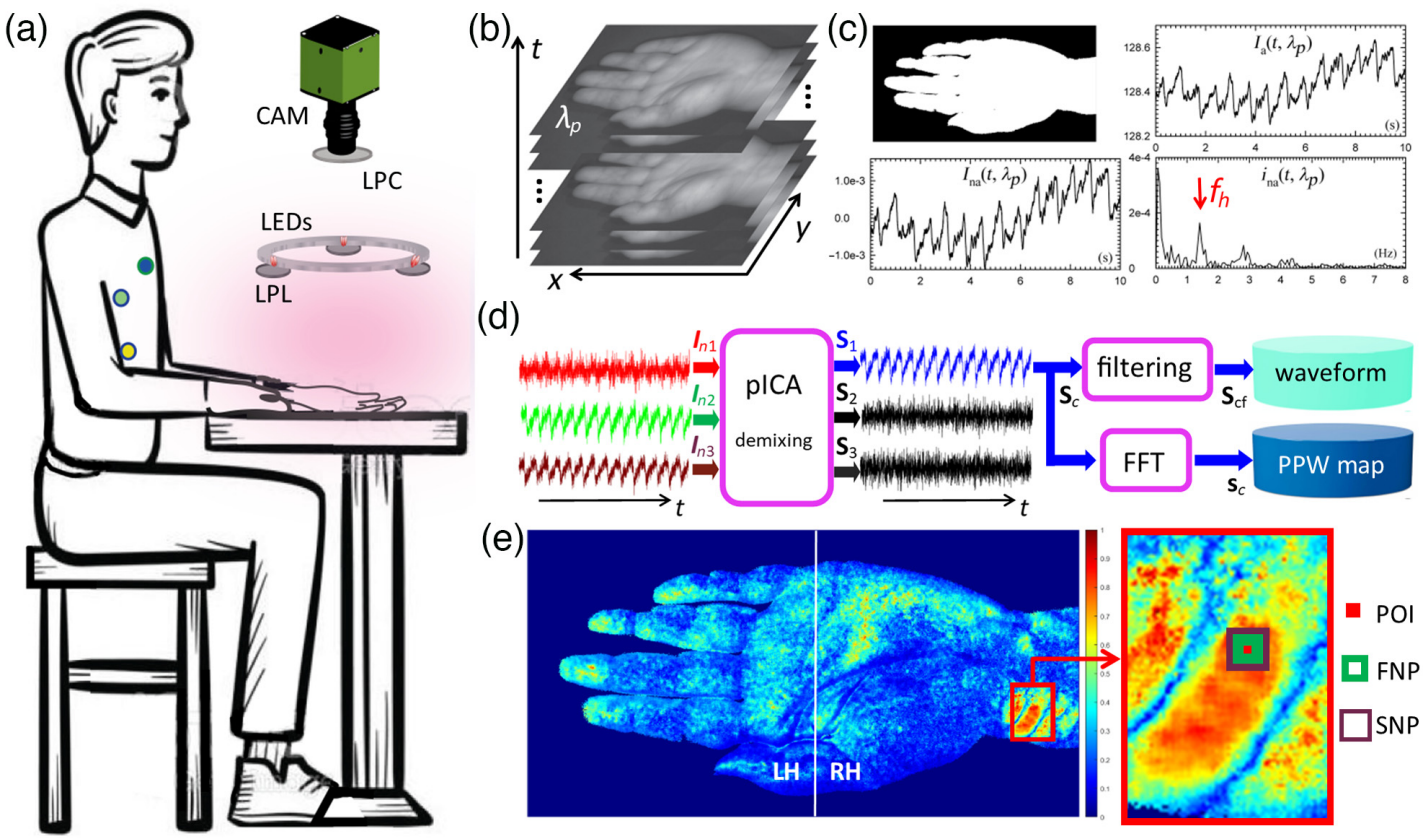
(a) iPPG imaging and ECG measurement: LEDs = LED arrays, CAM = camera, LPL(LPC) = linear polarizers for LEDs (CAM), three circular dots on chest indicating ECG electrode positions for data acquired with $\lambda_{p}=850\;\mathrm{nm}$. (b) Image stack. (c) Example of segmented image, time plots of averaged pixel intensity $I_{a}(t,\lambda_{p})$ over hand region, normalized intensity $I_{na}(t,\lambda_{p})$ and its Fourier transform $i_{na}(f,\lambda_{p})$ with heartbeat rate $f_{h}$ marked. (d) Work-flow chart for extracting PPW map and waveform. (e) Two choices of $I_{n2}$ and $I_{n3}$ as input signals for PFS scheme of single-pixel POI are shown in PPW map of volunteer #1 with $\lambda_{p}=850\;\mathrm{nm}$: with FNP and SNP in magnified view (right) and with LH and RH regions separated by white line (left).

Figure 1(a) illustrates the imaging configuration for acquisition of all iPPG stacks in 10 wavelength bands and ECG signals from three electrodes in some imaging sessions. As shown in Figs. 1(b) and 1(c), the heartbeat rate $f_{h}$ was determined from $i_{na}(f,\lambda_{p})$ as the Fourier transform of the hand-region averaged pixel intensity $I_{na}(t;\lambda_{p})$. The value of $f_{h}$ was validated against the value measured by the pulse oximeter and an acquired stack was discarded if they differ by >10%,^29^ which occurred mainly due to hand motion. With the PFS scheme for the input vectors to pICA, we obtained $S_{c}(x,y;\lambda_{p})$ for each pixel in the hand region followed by calculation of $r_{cf}(x,y;\lambda_{p})$ to determine a PPW map as illustrated in Fig. 1(d). To increase image contrast of these maps, two ways of forming the input vectors of $I_{n2}$ and $I_{n3}$ in the PFS scheme were compared: One with FNP and SNP and the other with pixel intensities averaged over left half-hand (LH) and right half-hand (RH) regions as illustrated in Fig. 1(e). The output vectors of pICA for the two approaches are compared with Fig. S2 of the Supplemental Material, which show significant reduction in the contrast of PPW maps. This is due to the dominance of $S_{\mathrm{cf}}$ by the input vectors formed by the pixels averaged over regions of LH and RH that contain much more pixels than those of FNP and SNP. The following results of synthesized PPW maps were obtained by the PFS scheme using the time vectors of POI, FNP, and SNP as the input vectors to pICA.

**Fig. 2:**
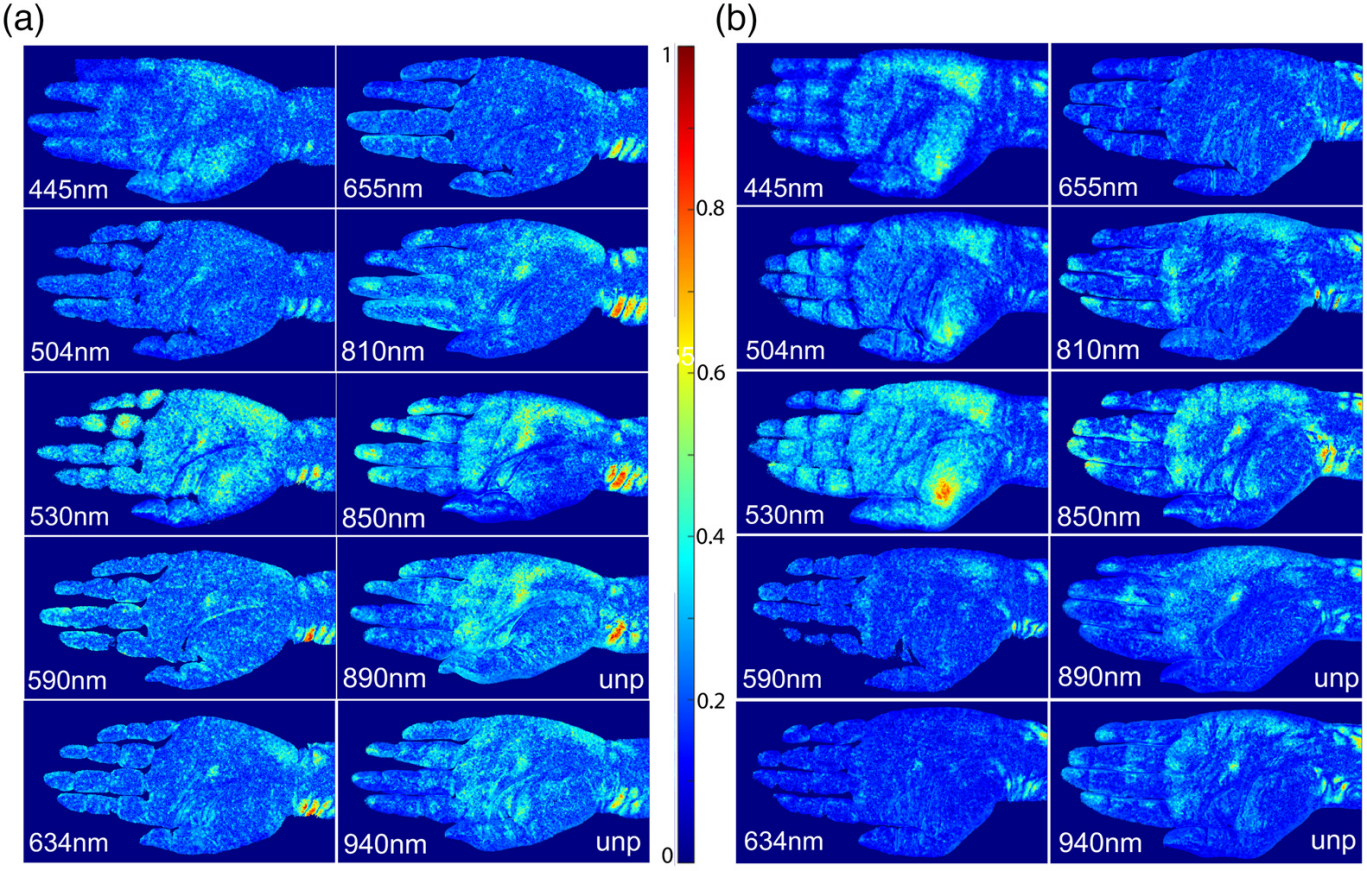
PPW maps of $r_{\mathrm{cf}}$ values acquired in 10 wavelength bands. All maps are normalized by the same maximum and minimum of pulse wave index values among all bands for each volunteer as indicated by the color bar with peak wavelength $\lambda_{p}$ marked on bottom of each map: (a) volunteer #1; (b) volunteer #2. Cross-polarized configuration was used for all maps except the two with $\lambda_{p\;}=890$ and 940 nm marked as unpolarized (unp).

Ten monochromatic iPPG stacks have been acquired from volunteers #1 and #2 with $\lambda_{p}$ from 445 to 940 nm using the cross-polarization configuration for study of the wavelength effect on consistency between synthesized PPW maps and palmar artery tree. Figure 2 presents the PPW maps by cross-polarized imaging except those of $\lambda_{p}=890$ and 940 nm. For these two bands, unpolarized imaging was also performed that yield PPW maps of higher contrasts by removing the polarizing films of low transmission. Figure S3 of the Supplemental Material includes the maps by cross-polarized imaging for all bands. All PPW maps were obtained by the PFS scheme with single-pixel POIs to preserve the high resolution of the iPPG stacks. Considerable similarity can be observed between the two volunteers on the distributions of pixels of high pulse wave index values for the bands of $\lambda_{p\;}=445$, 530, 810, 850 and 890 nm. Furthermore, these pixels appear to align with the anatomic structure of palmar artery.^30^^,^^31^

If one divides the PPW maps into two groups of visible and NIR by the wavelength of 700 nm, the visible group shows significantly smaller contrasts and pixel numbers of strong PPW signals. These differences can be clearly understood in terms of light absorption and scattering in skin tissues. For artery blood with red blood cells of nearly 100% oxyhemoglobins, the absorption coefficient $\mu_{a}$ oscillates across three orders of magnitude for light wavelength between 400 and 1000 nm, with maximum at the short end and minimum around 700 nm, while the scattering coefficient $\mu_{s}$ varies slowly in this range.^32^ In comparison, bloodless skin dermis absorb weakly, but its large value of $\mu_{s}$ decreases considerably for increasing wavelength from 400 to 700 nm and then remains fairly flat up to 1000 nm.^33^ The anisotropy factor g increases from 0.4 to 0.8 for bloodless skin dermis and varies from around 0.7 to above 0.9 for blood when wavelength increases from 400 to 1000 nm.^32^^,^^33^ *In vivo* measurements of transmission changes across rat’s skin tissues provide additional evidences, which show transmission peaks around 850 nm by an about 24-fold increase from 500 nm and an about threefold decrease to 1000 nm.^34^ Taken together, optimal probing of artery in the layers of skin dermis, subpapillary and cutaneous plexus requires proper penetration depth for light to reach and be backscattered from artery and surrounding tissues. High differential absorption between blood and skin tissues is also necessary to yield sufficient contrast for a PPW map. Hence, one may attribute the enhanced PPW signals in the NIR group to the increased penetration depth and differential absorption, which is especially the case for iPPG data acquired with $\lambda_{p}=850\;\mathrm{nm}$.

### 5.2 Analysis of Waveforms by the PZA Scheme with Zone-Averaged POIs

**Fig. 3:**
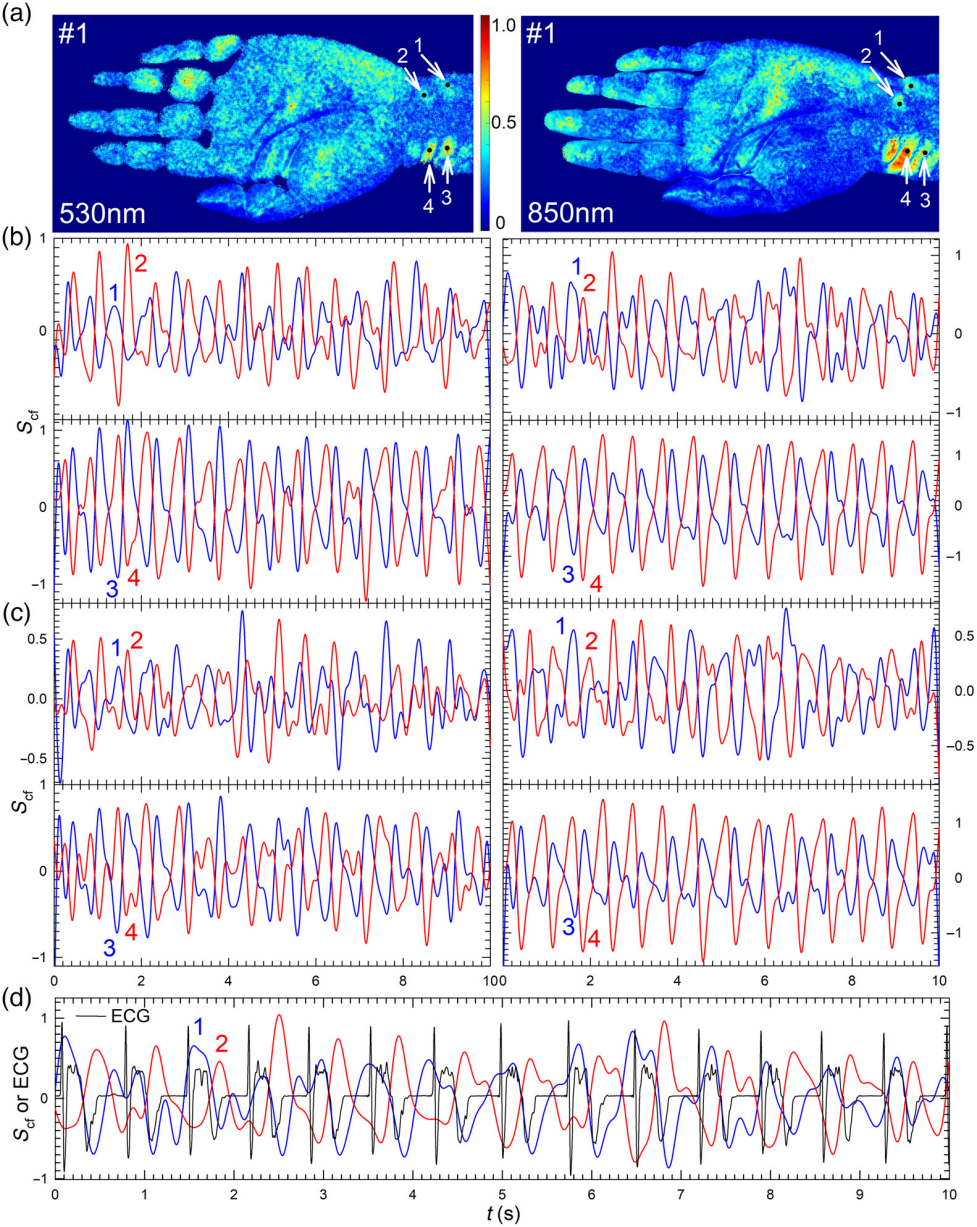
PPW maps and waveform data of volunteer #1 with $\lambda_{p\;}=530\;\mathrm{nm}$ (left column) and 850 nm (right column). (a) PPW maps by the PFS scheme with normalized values of $r_{\mathrm{cf}}$ as indicated by the color bar and POI sites marked by black dots and numbered; (b) waveforms of $S_{\mathrm{cf}}$ for zone-averaged POIs by PZA scheme with POI sites as marked; (c) waveforms of $S_{\mathrm{cf}}$ for single-pixel POIs by PFS scheme with POI sites as marked; (d) waveforms of ECG and $S_{\mathrm{cf}}$ for zone-averaged POIs by PZA scheme with POI sites as marked and $\lambda_{p\;}=850\;\mathrm{nm}$.

Here, we compare the waveforms at different POIs selected from the PPW maps of $\lambda_{p}=530$ and 850 nm. The input signal vectors of pICA were obtained by the PZA scheme with zone-averaged POI as $I_{n1}$ and larger zones for $I_{n2}$ and $I_{n3}$ to enhance SNRs in the PPW signals. Figure 3 compares the two schemes of PZA and PFS on four POI sites in the wrist region of volunteer #1. The mean value of SNR gain or *g*_SNR_ as defined in Eq. (4) increases to 4.31 by the PZA scheme in Fig. 3(b) from 3.93 by the PFS scheme in Fig. 3(c). To validate PPW signals extracted by pICA, we also plot in Fig. 3(b) the ECG waveforms measured simultaneously with iPPG imaging at $\lambda_{p}=850\;\mathrm{nm}$. The waveforms demonstrate unambiguously the synchronization between the two signals. The PPW maps and waveforms of 12 POI sites in the wrist and midpalm regions are shown in Fig. S4 of Supplemental Material, which were derived from the iPPG stacks of two volunteers acquired at $\lambda_{p}=445\;\mathrm{nm}$. These results show that combining zone averaging with pICA allows accurate extraction of PPW waveforms even in the case of shallow penetration for illumination of $\lambda_{p}=445\;\mathrm{nm}$.

**Fig. 4:**
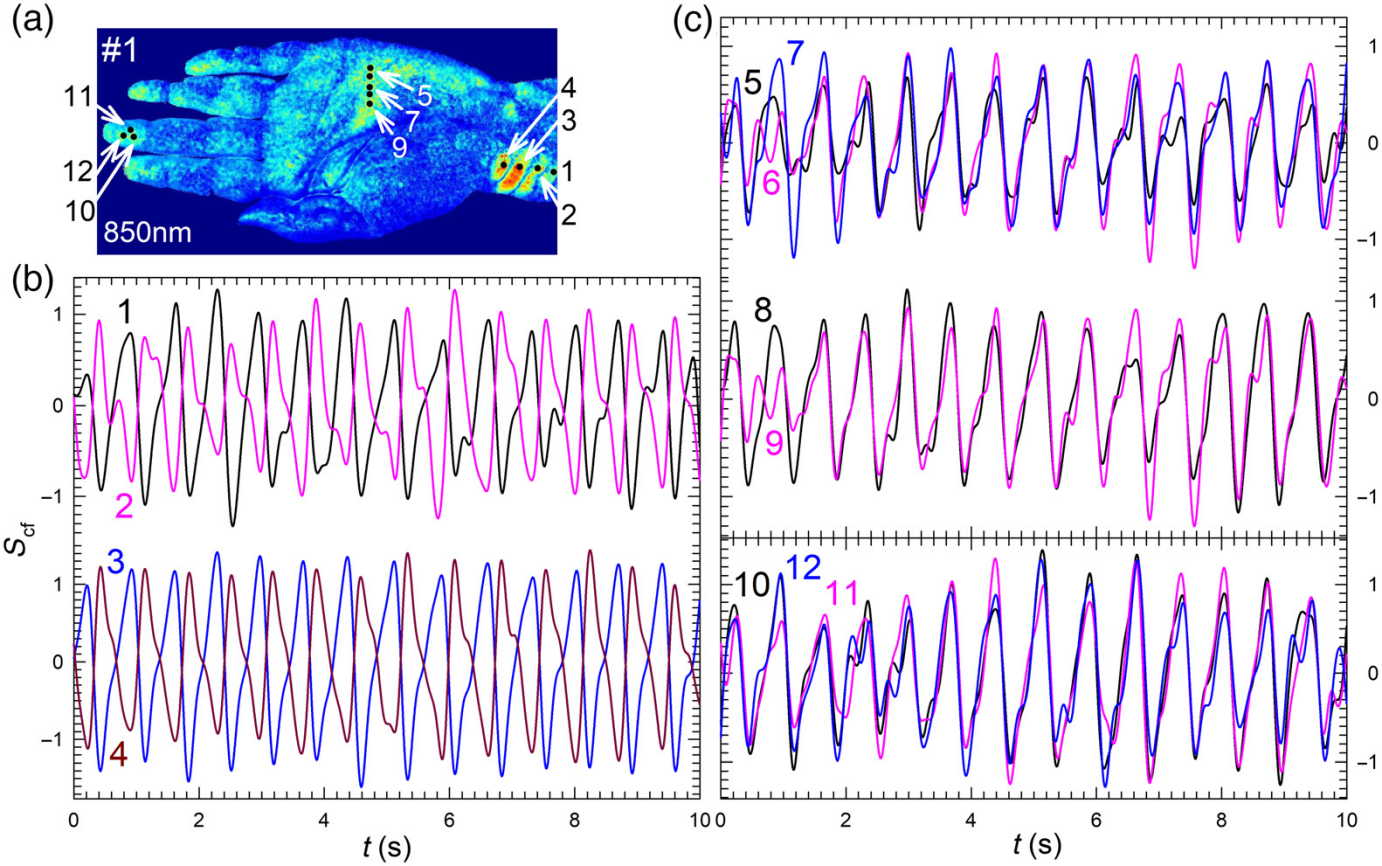
PPW map and $S_{\mathrm{cf}}$ waveforms of 12 POIs from iPPG data of volunteer #1 acquired at $\lambda_{p}=850\;\mathrm{nm}$. (a) PPW map with normalized values of $r_{\mathrm{cf}}$ as indicated by color bar and POIs marked by black dots and numbered; (b) waveforms at POIs in wrist region; (c) in midpalm and fingertip regions.

After comparison of PPW maps among the 10 wavelength bands, we chose those with $\lambda_{p}=850\;\mathrm{nm}$ to analyze the phase relations of waveforms given by $S_{\mathrm{cf}}(x,y;t;\lambda_{p})$ in three regions of wrist, midpalm and fingertip based on palmar artery anatomy. Figure 4 presents the PPW map of volunteer #1 with 12 POIs of strong PPW signals. With the PZA scheme, the first input to pICA calculation for a selected POI at (x,y) was obtained by a zone averaged value of pixel intensity over a 9×9 array centered on the pixel at (x,y). To assess the intensity gradient, we calculated the standard deviation of pixel intensity for each POI over its array in Fig. 4 and found its ratio to the averaged pixel intensity ranging from 1.7% to 2.4%. These results indicate that the zone for POI averaging in PZA scheme is sufficiently small to ensure the spatial resolution for comparing timing of PPW among neighboring POIs.

One may note from the peak and foot positions of waveforms in Figs. 4(b) and 4(c) that some neighboring POIs in the wrist region exhibit an out-of-phase (OOP) relation. The same OOP relation occurs among the POIs in the wrist region for data acquired with $\lambda_{p\;}=530$ and 445 nm as shown in Fig. 3 and Fig. S4 in Supplemental Material and other wavelength bands (not shown). In contrast, POIs in either midpalm or finger region exhibit in-phase or nearly overlapping waveforms. Because the speeds of PPW propagation in human artery are on the scales of about 1 to 10 m/s, one expects very small phase changes in the waveforms among neighboring POIs with distances less than or around 10 mm. This indicates that the above OOP relation is not due to propagation of PPW. The OOP relations have also been reported by others.^19^

To understand the phase relations, one needs to consider the effects of light wavelength and pulsatile oscillation of artery wall on extracted PPW signals in the form of $S_{\mathrm{cf}}(x,y;t;\lambda_{p})$. The former affects the optical parameters of blood and tissues and thus the depth of tissues interrogated by light while the latter leads to morphologic variations of artery and motion of surrounding tissues. Both alter the waveforms in an intertwined manner. As previously discussed, the values of optical parameters influence the probed volume of tissues and image contrast of PPW maps. But these differences should not lead to OOP relations in PPG waveforms. Otherwise, OOP relations should also occur on POIs outside the wrist region. One, therefore, needs to consider the morphologic changes and related motion in tissues surrounding artery with an oscillating wall, which leads to significant skin surface vibration if the artery is of relatively large size and close to skin surface. Indeed, the surface vibration in the wrist region can be observed by naked eyes and has been previously measured.^23^ As the interface with the largest mismatch in refractive index, the skin surface contributes significantly to the scattered light detected by camera and thus can vary the timing or phase of PPW signals from the measured iPPG data in certain wrist locations. For other hand regions, the surfaces vibration appears negligible due to either large depth of artery from the surface in the case of midpalm or small volume changes of arterioles in the case of fingertip. We thus conclude that the OOP relations appearing at some POIs in the wrist region of iPPG data is a result of skin surface vibration by PPW.

### 5.3 Measurement of Pulse Wave Velocity

**Fig. 5:**
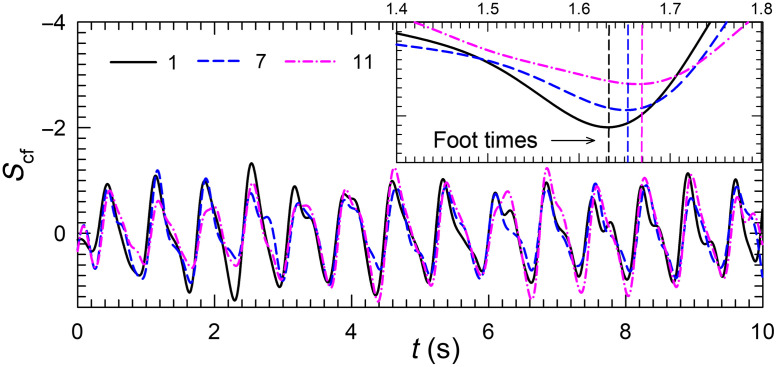
$S_{\mathrm{cf}}$ waveforms of three POIs shown in Fig. 4(a) on inverted scale of volunteer #1 with $\lambda_{p\;}=850\;\mathrm{nm}$. Inset: vertical dash lines indicate foot times of three POIs before systolic peaks on an expanded time scale.

We selected 10 out of the 12 POIs in three regions of wrist, midpalm, and fingertip as shown in Fig. 4 to determine the mean value of delay times between waveforms. Two POIs in the wrist region were excluded for their OOP phase relations in comparison to other POIs. Figure 5 presents examples of $S_{\mathrm{cf}}$ waveforms for POIs marked as 1, 7, and 11 in Fig. 4(a) on an inverted vertical axis. The previously discussed foot times before the systolic peaks were first used as the time markers for calculation of delay times or $\tau (r,r^{\prime };c)$ as illustrated in Fig. 5. The mean delay times denoted as $\tau_{m}(r,r^{\prime })$ in Eq. (5) were then obtained by averaging $\tau (r,r^{\prime };c)$ over all cardiac cycles to determine PWV values between two regions of r and $r^{\prime }$.

**Fig. 6:**
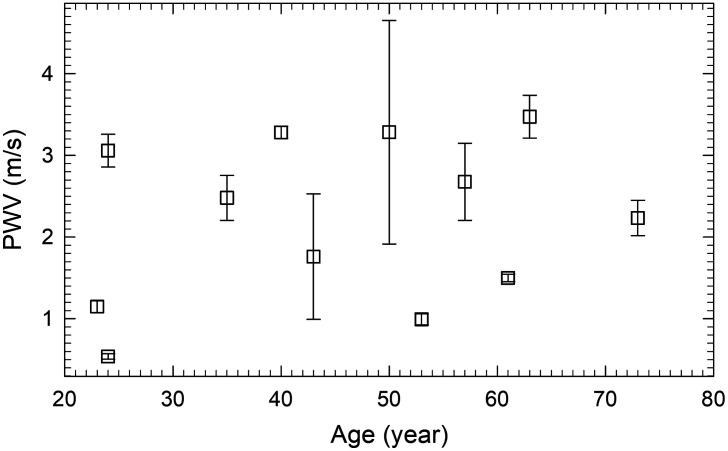
PWV values of 12 volunteers determined from iPPG data with $\lambda_{p\;}=850\;\mathrm{nm}$. The symbols and error bars represent the mean and standard deviation values of three PWV values between two regions of wrist and midpalm, midpalm and fingertip, and wrist and fingertip.

Figure 6 presents the mean and standard deviation of PWV values for three paired regions from wrist to midpalm, from midpalm to fingertip and from wrist to fingertip of the 12 volunteers versus their ages. We note that the estimation of PWV values by Eq. (5) tends to underestimate since $d(r,r^{\prime })$ given by the straight-line distance is shorter than the actual distances of PPW propagates in palmar artery. Rather, the PWV values in Fig. 6 are intended to demonstrate the potentials of the proposed iPPG method here for determination of time delays and PWV in peripheral arteries of the hand. The PWV values reported here are smaller than those of the aorta and its first branches such as carotid, femoral, and brachial arteries with larger diameters in a range of 3 to 12 m/s,^35^^,^^36^ which may be attributed to differences in vessel size, blood pressure gradient from brachial to finger and wave reflections from downstream branches in palmar artery tree.^37^ We would also like to note that the measured PWV values in this study agree well with those measured with closely placed PPG sensors on carotid artery.^38^

As a versatile platform, the iPPG technology has the capacity to become a cost-effective tool for monitoring blood flow and cardiac activities. Yet, its potentials to detect PPW and PWV for assessment of artery wall conditions remains to be investigated and fulfilled. To achieve this goal, we have developed an innovative algorithm by combining ICA and spatiotemporal filtering for extraction of weak PPW signals from monochromatic iPPG data. Different schemes of forming input vectors for pICA were explored and two were employed to derive PPW maps by the pulse wave index $r_{\mathrm{cf}}$ and determine time delays among POIs of strong PPW signals. Quantitative PPW maps presented in Fig. 2 in 10 wavelength bands makes it possible to find optimized wavelength band of $\lambda_{p}=850\;\mathrm{nm}$ for probing PPW. The phase relations of waveforms are difficult to interpret since PPW modulates the morphology of both arteries and surrounding tissues that include the orientation of skin surface due to vibration in regions with large and superficial arteries. By accurate determination of $S_{cf}$ at multiple POIs and compare these in different wavelength bands, shown in Figs. 3 and 4, we have demonstrated that the strong surface vibration by PPW in the wrist region causes the OOP relations in waveforms at certain POIs. These results are critical for the selection of POIs to compare and analyze phase variations of the waveforms presented in Fig. 5. Only with this insight, it became possible to identify the related foot times among POIs in different hand regions per cardiac cycle and correctly determine their time delays for estimation of PWV values. Taken together, these results demonstrate the capabilities of the iPPG methods to develop biomarkers for quantifying wall stiffness of superficial arteries and assessing risks of cardiovascular events. To evaluate the clinical potentials of the iPPG method, it is important to note the limitation of the method due to the small penetration depths of visible and NIR light in human skin tissues. Furthermore, a clear understanding of the advantages and limitations of the iPPG method requires quantitatively modeling of fluid dynamics in artery blood and tissue optics underlying feature extraction from scattered light signals.

## 6 Summary

We have shown an iPPG method that allows the acquisition of an image stack within 10 s and retrieval of map and waveform data of PPW in superficial palmar artery with monochromatic LED illumination. With a novel and robust pICA algorithm, the cardiac component of the iPPG data can be extracted with high spatial resolution to select POIs of strong PPW signals for waveform analysis. With an optimized illumination wavelength band around 850 nm, PPW waveforms among multiple locations near the palmar artery have been analyzed and the PWV values of 12 healthy volunteers without cardiovascular diseases and hypertensions have been estimated. These results demonstrate the capability of the presented method in the quantification of PPW propagation in superficial arteries and assessment of artery wall stiffness and other conditions.

## Code, Data, and Materials Availability

Code, data and materials associated with this article are available upon request sent to the corresponding author.
