# Supplementary material for: Photoplethysmographic imaging and analysis of pulsatile pressure wave in palmar artery at 10 wavelengths
Source: J Biomed Opt. 2022 Nov 10;27(11):116004. doi: 10.1117/1.JBO.27.11.116004 (PMC9647835; doi:10.1117/1.JBO.27.11.116004)
Supplement: Supplementary file 1 [file JBO_027_116004_SD001.pdf]

Supplemental Material for

Photoplethysmographic imaging and analysis of pressure wave in palmar artery at ten wavelengths

Jiahong Jin, Jun Q. Lu, Cheng Chen, Ruihai Zhou, and Xin-Hua Hu

S1. Materials and Methods

We have developed 10 units of LED based illumination with peak wavelength values of λ_p ranging from 445 to 940 nm. The model numbers and manufacturers of LEDs are listed in Table S1 below.

Table S1. Model numbers and manufacturers of LEDs for iPPG illumination

λ_p (nm)	450	504	530	590	634	655	810	850	890	940
Model #	GD CSSR M2.14-ARAT-24-1	L1CU-CYN10000000 00	LZ1-00G102 -0000	LCY CLBP-KZLY-5F5G-8E8G	GR CS8PM 1.23-JUKQ-1	GH CSSRM 2.24-VLVN-1-1	SFH 4780S	LTE-R38386 A-ZF-U	VSMF37 10-GS18	LTE-R38381 S-ZF-U
Manufac-turers	Osram	Lumile ds	Osram	Osram	Osram	Osram	Osram	Lite-On	Vishay	Lite-On

Figure S1 illustrates the iPPG system developed for simultaneous acquisition of iPPG data from a hand and ECG signals with 3 ECG electrodes placed on chest of a volunteer.

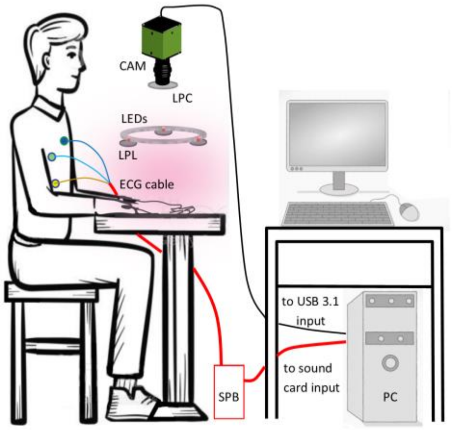

Fig. S1 The schematic of system for simultaneous acquisition of iPPG data and ECG signals from a volunteer: LEDs=LED arrays, CAM=camera, LPL(LPC)=linear polarizers for LEDs (CAM), SPB=signal processing board for ECG signals with 3 circular dots representing the ECG electrodes.

S2. Results

In Fig. S2, we compare the two different choices of input vectors $\mathbf{I}_{n2}$ and $\mathbf{I}_{n3}$ for pICA calculations on the same selected pixel of interest (POI) whose time vector is used as the first input vector of $\mathbf{I}_{n1}$, which are shown as the top row in (A) and (B). In Fig. S2(A) the time vectors averaged over first-nearest-neighbor pixels (FNP) and second-nearest-neighbor pixels (SNP) vectors are defined as $\mathbf{I}_{n2}$ and $\mathbf{I}_{n3}$ respectively while in Fig. S2(B) the time vectors averaged over half-hand pixels (LHP) and right half-hand pixels (RHP) are defined as $\mathbf{I}_{n2}$ and $\mathbf{I}_{n3}$. The first output IC assigned as $\mathbf{S}_{cf}$ or cardiac component (top row in middle column) shows much smoother time dependence in Fig. S2(B) than Fig. S2(A) because of the much smoother time vectors of the LHP and RHP used as $\mathbf{I}_{n2}$ and $\mathbf{I}_{n3}$. This consequently reduce the image contrast of the synthesized PPW map derived from $\mathbf{S}_{c}$ at different pixels. In the right column of Fig. S2, we have also shown the ECG signals in the top row which demonstrate the extracted PPG signals of $\mathbf{S}_{cf}$ are synchronous to the ECG or cardiac signals.

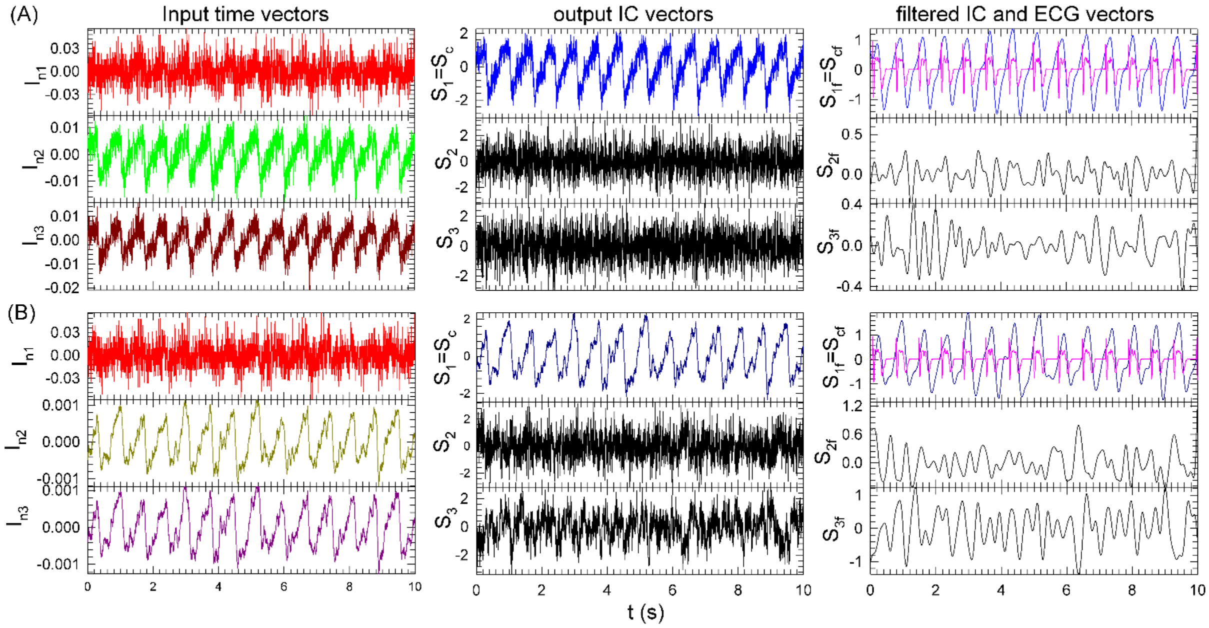

Fig. S2 Comparison of two choices of $\mathbf{I}_{n2}$ and $\mathbf{I}_{n3}$ as input vectors to pICA in addition to $\mathbf{I}_{n1}$ for POI by the PFS scheme. Also displayed are the corresponding output ICs of $\mathbf{S}_{1f}$(=$\mathbf{S}_{cf}$), $\mathbf{S}_{2f}$ and $\mathbf{S}_{3f}$ after frequency filtering in time domain with λ_p = 850 nm. (A) $\mathbf{I}_{n2}$ and $\mathbf{I}_{n3}$ are given by the average pixel intensity of FNP and SNP. (B) $\mathbf{I}_{n2}$ and $\mathbf{I}_{n3}$ are given by those of LH and RH. The pink lines in the right columns represent the simultaneously acquired ECG signals

The PPW maps with cross-polarized iPPG stacks acquired from volunteers #1 and #2 in all 10 wavelength bands are presented in Fig. S3. The lack of image contrasts in the maps of λ_p = 890 and 940 nm is due to the reduced LED irradiance on hand by the low transmission of the polarizing films at these wavelengths.

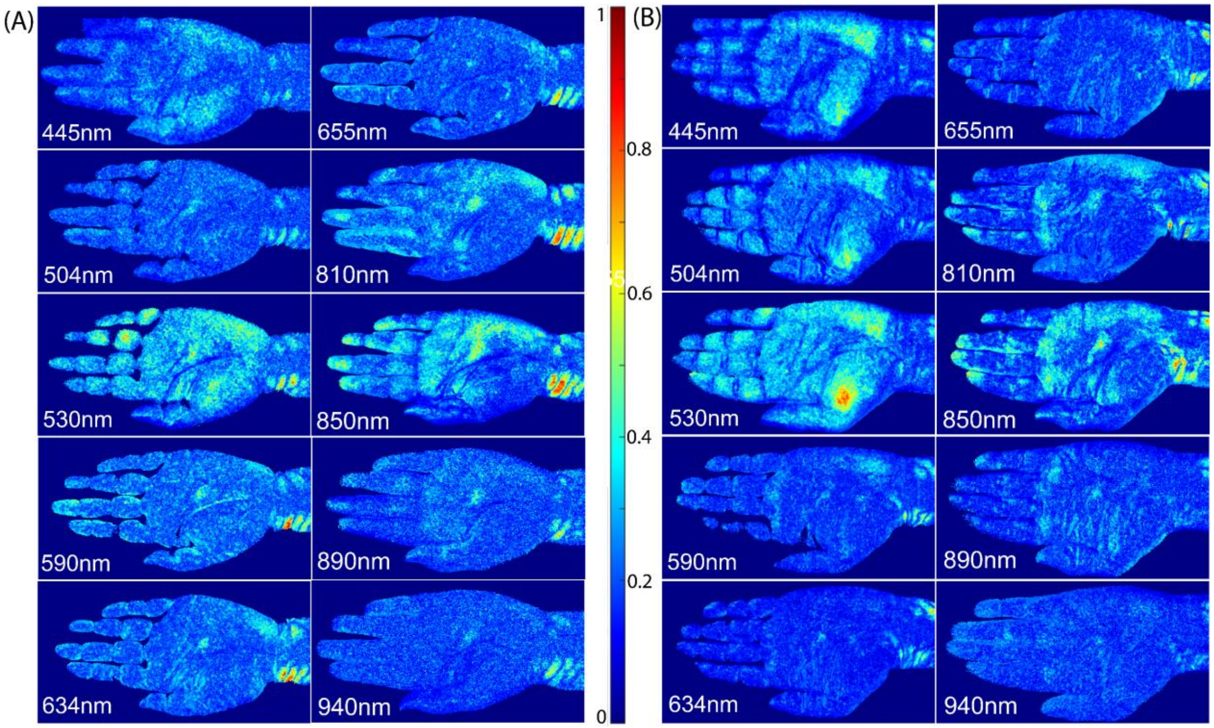

Fig. S3 The PPW maps of 2 volunteers with normalized r_{cf} values as indicated by the color bars and values of λ_p marked on bottom of each map: (A) volunteer #1 and (B) volunteer #2. All imaging session were performed in cross-polarized configuration.

Fig. S4 presents the PPW maps of 2 volunteers to demonstrate that the PPG waveforms can be extracted by the PZA scheme of zone-averaged POIs even in the case of relatively weak PPG signals with λ_p = 445 nm. Twelve sites were selected from each volunteer's map along a path based on the anatomy of palmar arch, radial and ulnar artery. Despite the relatively large noise, the waveforms show clearly cardiac cycles driving PPW which are evidently distinguishable between the two volunteer and useful for health monitoring, biometric identification and diagnosis of diseases at this and other wavelengths. For example, the waveforms of volunteer #1 present higher values of $r_{cf}(x, y; \lambda_p)$ at the sites of 11 and 12 near the radial artery of the wrist than those of 1 and 2 near the ulnar artery side. The relation reverses for the volunteer #2 which can also be seen in the PPW maps obtained at other visible wavelengths between the two volunteers.

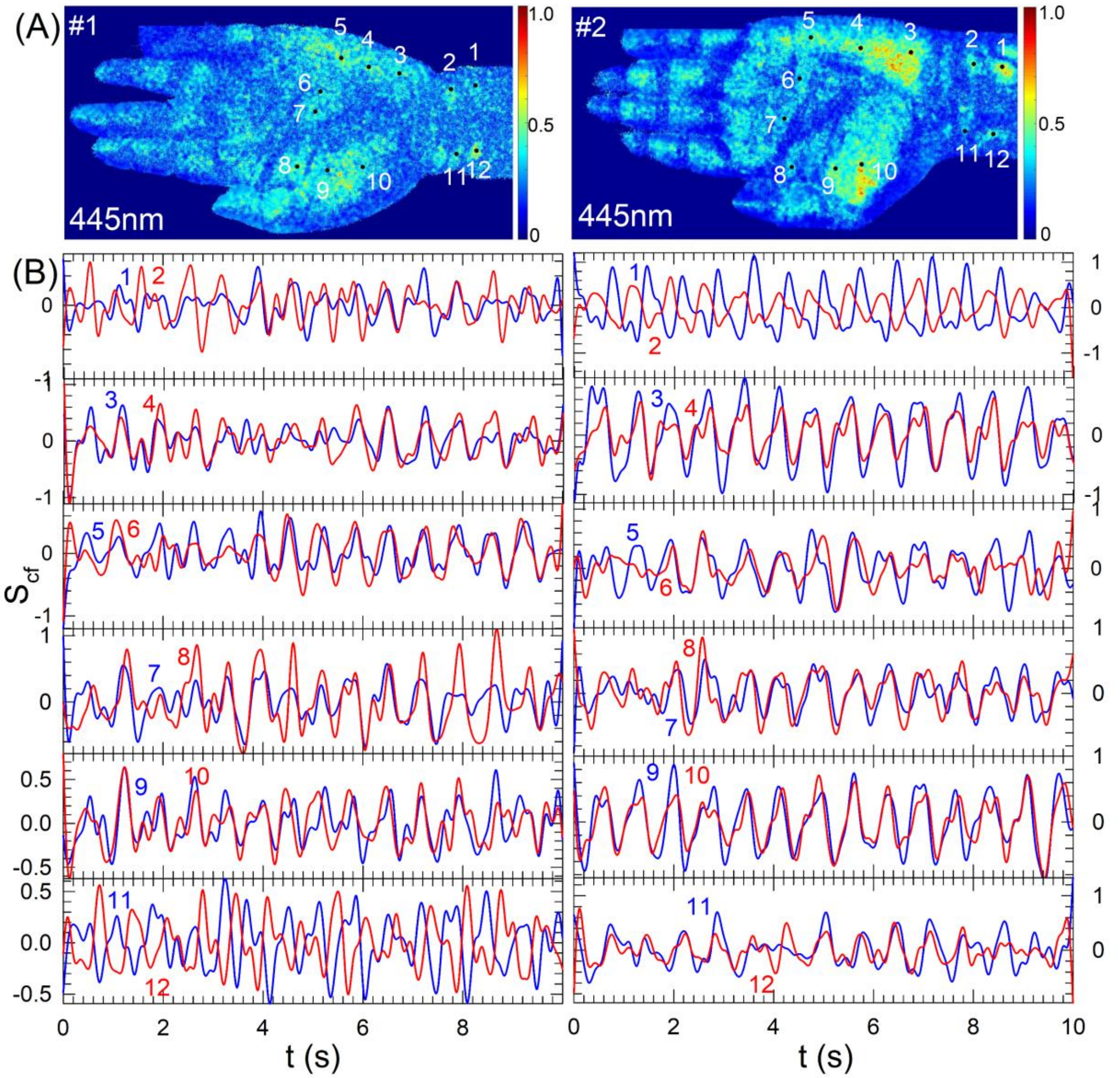

Fig. S4. The PPW maps and waveforms of 12 POIs of volunteer #1 (left column) and #2 (right column) in the band of λ_p=445nm at 12 POI sites: (A) PPW maps by the PFS scheme with POIs marked by black dots and numbered; (B) waveforms of zone-averaged POIs by the PZA scheme.
